# Multi-Source Data Fusion and Hydrodynamics for Urban Waterlogging Risk Identification

**DOI:** 10.3390/ijerph20032528

**Published:** 2023-01-31

**Authors:** Zongjia Zhang, Yiping Zeng, Zhejun Huang, Junguo Liu, Lili Yang

**Affiliations:** 1 School of Environment, Harbin Institute of Technology, Harbin 150001, China; 2 Department of Statistics and Data Science, Southern University of Science and Technology, Shenzhen 518055, China; 3 School of Environmental Science and Engineering, Southern University of Science and Technology, Shenzhen 518055, China; 4 Henan Provincial Key Laboratory of Hydrosphere and Watershed Water Security, North China University of Water Resources and Electric Power, Zhengzhou 450046, China

**Keywords:** risk identification, disaster risk assessment, urban waterlogging, multi-source data, environmental risk, flood prediction, hydrodynamics, GIS

## Abstract

The complex formation mechanism and numerous influencing factors of urban waterlogging disasters make the identification of their risk an essential matter. This paper proposes a framework for identifying urban waterlogging risk that combines multi-source data fusion with hydrodynamics (MDF-H). The framework consists of a source data layer, a model parameter layer, and a calculation layer. Using multi-source data fusion technology, we processed urban meteorological information, geographic information, and municipal engineering information in a unified computation-oriented manner to form a deep fusion of a globalized multi-data layer. In conjunction with the hydrological analysis results, the irregular sub-catchment regions are divided and utilized as calculating containers for the localized runoff yield and flow concentration. Four categories of source data, meteorological data, topographic data, urban underlying surface data, and municipal and traffic data, with a total of 12 factors, are considered the model input variables to define a real-time and comprehensive runoff coefficient. The computational layer consists of three calculating levels: total study area, sub-catchment, and grid. The surface runoff inter-regional connectivity is realized at all levels of the urban road network when combined with hydrodynamic theory. A two-level drainage capacity assessment model is proposed based on the drainage pipe volume density. The final result is the extent and depth of waterlogging in the study area, and a real-time waterlogging distribution map is formed. It demonstrates a mathematical study and an effective simulation of the horizontal transition of rainfall into the surface runoff in a large-scale urban area. The proposed method was validated by the sudden rainstorm event in Futian District, Shenzhen, on 11 April 2019. The average accuracy for identifying waterlogging depth was greater than 95%. The MDF-H framework has the advantages of precise prediction, rapid calculation speed, and wide applicability to large-scale regions.

## 1. Introduction

Urban waterlogging disasters are characterized by high suddenness, extensive coverage, and catastrophic devastation, and have become one of the most threatening disasters in the world [1]. In recent decades, continued urbanization has resulted in an annual increase in the population density and land use [2]. Numerous flood-prone low-lying sites have been incorporated into building plans, and poor drainage capacity has further exacerbated the risk of flooding [3]. During extreme rainstorms or typhoons, the intensity of the storm often far exceeds the design drainage capacity of the municipal system [4]. The surface rainfall cannot be drained in a timely manner, thus forming surface runoff and further converging into waterlogging. Urban flooding risk identification can predict the area, depth, and velocity of waterlogging under different storm intensities and generate real-time urban storm flooding risk maps. This can be used as a decision-making resource for predicting urban floods during extreme rainfall occurrences [5]. Early warning information has been a crucial component of flood mitigation [6]. There has been a lot of research conducted on urban waterlogging prediction and risk identification. The urban waterlogging prediction methods can be roughly divided into five categories: statistical methods, hydrodynamic methods, data-driven approaches, remote sensing methods, and multi-source data fusion methods.

Statistical methods. The moving-window variance technique, which does not require baseflow estimation for defining the start and end of a flood event, was suggested [7]. In flood vulnerability mapping, the robustness of the statistical and MCDM (multi-criteria decision making) models is evaluated. The validation results demonstrate that the statistical models provide more accurate predictions than the MCDM model [8]. To assess urban waterlogging risk, a spatial framework integrating WNB (Weighted Naive Bayes) with GIS was developed, and its results demonstrated a more accurate spatial pattern of the urban waterlogging risk [9]. A flood risk detection method that uses static Bayesian networks and historical data to generate flood risk nodes can explain the flood prediction logic more clearly than the machine learning “black box” model [10].

Hydrodynamic methods. The hydrodynamic model necessitates extensive urban subsurface data, and for data-rich study areas, the hydrological model can produce more accurate simulation results. The disadvantage is that the modeling process is complex, so it is difficult to apply it to large-scale urban flooding risk identification. A methodology considering the variability of the building types and the spatial heterogeneity of land surfaces was proposed. The model complexity is increased stepwise by adding components to an existing 2D overland flow model [11]. Some research analyzed local rainfall patterns and used a coupled hydrodynamic model for the waterlogging simulation [12]. Using gradient estimation, the method based on the D8 algorithm and fourth-order finite-difference techniques has been shown to locate the runoff and ponding points [13].

Data-driven approach. Chen et al. tested and compared the prediction capabilities of the naive Bayes tree (NB Tree), the alternating decision tree (AD Tree), and the random forest (RF) approaches for the spatial prediction of flood. The probability certainty factor (PCF) technique was utilized to assess the relationship between the factors and flood occurrences [14]. The long and short-term memory and support vector regression models perform better than the artificial neural network models for hourly flood forecasting at a fine scale. Comparing the models during the dry season and the rainy season revealed that the models were more sensitive during the rainy season. It was found that the sensitivity of the models was higher in the rainy season [15]. Flood data has the advantage of being easy to call when analyzing floods. Some scholars have used GBDT to build a model for predicting flood zones and can achieve an accuracy of 88.48%, which greatly increases the prediction performance [16]. The prediction results of machine learning flood identification methods are accurate, but the model is difficult to interpret. On the other hand, the method requires high requirements for historical flooding data, and good training results often need a large amount of spatial and temporal flooding data to support them. In reality, it often relies on the deployment of a large number of sensors to collect the spatial and temporal flooding data, which is relatively costly.

Remote sensing methods. With the gradual rise in remote sensing resolution, the inundation extraction model based on remote sensing has been able to be employed for inundation risk identification. This method mainly compares the remote sensing images before and after flooding in a particular area and extracts the extent and depth of the inundation after processing. It can provide pre-disaster reference information as well as statistics about the affected area. Shao et al. put forward an urban multi-level watershed runoff monitoring model that can be used to analyze the relationship between impervious surfaces and urban runoffs at multi-scale watersheds [17]. A hybrid method for urban flood mapping by combining random forest and texture analysis based on high-resolution UAV imagery was proposed. A random forest consisting of 200 decision trees was utilized to extract the inundated areas [18]. The remote sensing methods lack a description of the flooding process and cannot be continuously simulated in real time. Resources, such as remote sensing satellites and aircraft, are scarce and unevenly distributed, making it difficult to promote their use on a large-scale in a short period of time.

Multi-source data fusion methods. The city waterlogging application domain extension (CTVLADE) includes data from hydrology, meteorology, planning, and mapping to study the mechanisms of waterlogging occurrence and cessation [19]. A novel emergency decision model based on a similarity algorithm for case inference is created and validated using the emergency case of urban waterlogging. It is proved that the model has a high degree of adaptability and can provide a good reference to assist the decision making for disaster incidents [20]. Li et al. combined multi-source data fusion and neural network modeling to build a prediction model. By constructing a fuzzy matrix, they achieved the goal of a low error rate and faster computing speed [21].

In summary, the main problems of the current research include: (1) Inadequate description of the mechanism of urban waterlogging runoff yield and flow concentration, and an approach that lacks an explanation and simulation of the process and outcomes of floods. (2) Inadequate consideration of the factors influencing the formation and spread of urban waterlogging, as well as a significant gap between some assumptions and the actual situation. (3) The data pre-processing of the model is not meticulous, and the data’s generalizability and applicability are lacking. (4) Some strategies struggle to simultaneously improve the model’s prediction accuracy and processing efficiency. (5) It is unsuitable for large-scale regions.

This paper focuses on the identification of the waterlogging risk in large-scale urban areas. The main feature of this method compared to the above is that it can be applied to large-scale study areas. The modeling workload is smaller because there is no need to construct surface and underground pipe network models. The model parameters are easier to set, and the requirements for urban subsurface and historical waterlogging data are low. The calculation speed is faster and the accuracy is higher. Combined with the evolution simulation of hydrodynamics, the model is able to simulate the waterlogging process well and has good interpretability. Compared with other methods for the large-scale identification of urban waterlogging (Table 1), the method proposed in this paper has obvious advantages, especially for the large-scale study area, in terms of adaptability, computational speed, and interpretability (more △ Positive indicators). It is also the least dependent method on historical data (○ Negative indicators are lower).

## 2. Methodology

### 2.1. MDF-H Urban Waterlogging Risk Identification Framework

Multi-source data fusion is the process of combining data from different sources to evaluate the robustness and completeness of a given system. Since a single data source cannot provide enough information to fully detect a complex system, multi-source data fusion can yield comprehensive and reliable results after combining data sets that reflect various characteristics of the system [22]. According to the classification of algorithm concepts, there are three main categories, namely: physical model-based, parameter-based, and epistemic model-based, among which the parameter-based methods are most widely used.

**Figure 1:**
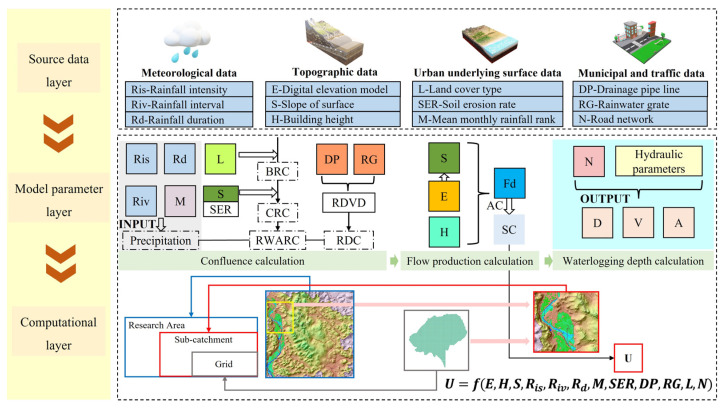
MDF-H Framework: where *U* is the unified fusion parameter, *E* is the elevation, *R_is_* is the rainfall intensity, *R_iv_* is the rainfall interval, *R_d_* is the rainfall duration, *H* is the building height, *S* is the slope, *F_d_* is the flow direction, *L* is the land cover type, *N* is the road network, *DP* is the drainage pipes, *RG* is the rainwater grate, *SC* is the sub-catchment, *M* is the mean monthly rainfall rank, *SER* is the soil erosion rate, *BRC* is the basic runoff coefficient, *CRC* is the comprehensive runoff coefficient, *RWARC* is the regional weighted mean runoff coefficient, *RDC* is the regional drainage coefficient, *D* is the waterlogging depth, *V* is the runoff flow velocity, and *A* is the area of *SC*. The data descriptions and sources used in this framework in the case study refer to Table 2.

This paper proposed a framework for urban flooding risk identification by combining multi-source data fusion and hydrodynamic modeling (Figure 1). We have combined the domain knowledge, references to the other literature, and simulation experiments to validate the selection and construction of the factors. The mechanism behind these factors is the process of the runoff yield and flow concentration formation due to waterlogging caused by heavy rainfall. Although this process is continuous and complex, we discretize the continuous confluence process and perform hydraulic calculations by dividing the computational unit. From the grid level to the sub-catchment level, the aggregation from micro to macro computation is realized. At the global scale of the study area, it is equivalent to a continuous process. The formation of urban storm waterlogging is related to several factors [23], which can be summarized into three main categories, namely gain factors, impedance factors, and flow concentration distribution factors. The gain factor is mainly the rainfall intensity and rainfall duration, which show a positive correlation with the scale of flooding. The return period of the rainfall refers to the average time between the possible occurrence of a rainfall intensity greater than or equal to this value, in years, and the recurrence period is inversely proportional to the frequency. The impedance factor co sist of certain factors that are negatively related to the formation of waterlogging and can impede the generation of standing water. The main factors include the surface infiltration capacity, drainage capacity, evaporation, etc. [24]. The flow concentration distribution factor is mainly related to topography, geomorphology, roads, and surface structures [25]. The rainfall causes a continuous increase in the surface runoff, and the overflowing surface runoff is influenced by gravity to flow horizontally from high terrain to low terrain areas. The continuously increasing surface runoff cannot be discharged by the drainage system in time, thus forming waterlogging in low-lying areas. In terms of the rainfall process, the extent and depth of waterlogging generally increases continuously. The specific process by which rainfall is eventually transformed into waterlogging is complex. The process can be divided into two phases based on hydrodynamics: runoff yield and flow concentration. The runoff yield is the flow of the rainfall falling to the ground, minus the infiltration, evaporation, and entry into the drainage network. The surface runoff formed in the runoff yield stage will be influenced by the topography and slope, from high terrain to low terrain. In the flow concentration stage, the continuous flow converges toward the lowest point of the area and forms internal flooding in a short period of time. The contribution of each factor to the formation of inland flooding is relatively independent, and we also performed correlation degree detection in certain regions without the problem of multicollinearity. It proves that there is no redundancy in the overall framework.

### 2.2. Building Multi-Source Parameter Layers

**Figure 2:**
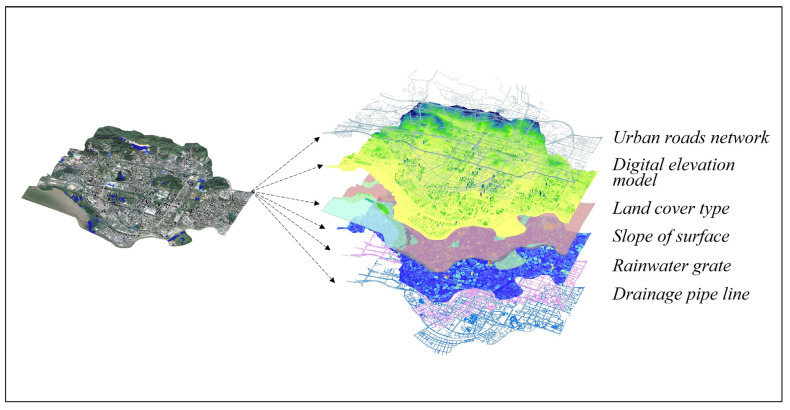
Building a multi-source data layer for a unified region (schematic diagram of the method).

Different parameter layers are constructed by combining the magnitudes of each influencing factor parameter and converting them into the form of parameters that can be involved in hydrological calculations. There are relatively large differences in the structure, format, and magnitude of each raw dataset. The DEM, land cover type, slope, etc. are raster data. The drainage pipes and rainwater outfalls are vector data, and rainfall data are time series. According to the final task objective, each type of data is unified into different parameter layers according to the study area (Figure 2), and the parameter information within each parameter layer is used as the input factor of the model.

### 2.3. Runoff Yield Simulation and Calculation

**Figure 3:**
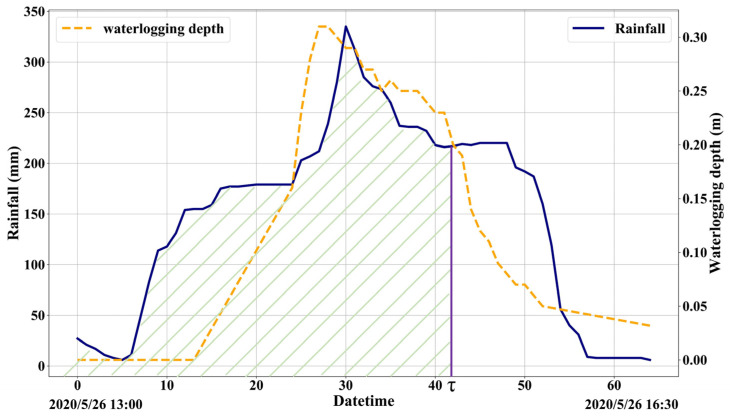
Rainfall process curve and depth of waterlogging (green diagonal part is the cumulative rainfall).

The model uses a variable rainfall input model in the runoff yield calculation. The Chicago rainfall model is based on the statistical storm intensity formula to design a typical rainfall process. The rainfall ephemeral time series is divided into two parts, pre-peak and post-peak, by introducing a rainfall peak coefficient to describe the moment when the peak of the rainstorm occurs [26]. According to the localized rainfall intensity formula, which can reflect the pattern of rainfall intensity over time [27], the Chicago rainfall model is generated for a specific rainfall recurrence period and rainfall peak coefficient. The rainfall time node parameter τ is introduced to quickly locate the cumulative rainfall and rainfall intensity at a certain moment (Figure 3).

#### 2.3.1. Time-Varying Rainfall Input Factor

According to the storm intensity Equation (1).

(1) $$q=\frac{167A\left(1+Clga\right)}{{\left(t+b\right)}^{n}}$$

where *q* is the rainfall intensity; *a* is the return period of storm; *t* is the rainfall duration; *A*, *C*, *b*, *n* are the rain force formula parameters.

Then the cumulative rainfall *R* at a moment *τ* is Equation (2).

(2) $$R=\int_{0}^{\tau }qTdT$$

where, *q* is the instantaneous rainfall intensity, *T* is the calculation time, and *τ* is the calculation moment.

(3) $$\varphi =\frac{Q_{r}}{Q_{q}}$$

The runoff coefficient is widely used in hydrological studies to characterize the proportion of the rainwater converted to the surface runoff. Where the runoff coefficient is widely used in hydrological studies to characterize the proportion of rainwater converted to the surface runoff, $Q_{r}$ is the converted surface runoff flow rate (m^3^/s), and $Q_{q}$ is the rainfall flow rate (m^3^/s) (Equation (3)). Within the urban area, land cover types can be divided into permeable and non-permeable surfaces. The permeable surface mainly includes forest land, grassland, irrigated land, wetland, gravel land, and bare soil. Non-permeable surfaces mainly include roads, building plots, rooftops, squares, parking lots, and other built-up land. The runoff coefficients of the two differ greatly [28]. The runoff coefficient not only varies depending on the type of land cover but is also influenced by the current water content in the surface medium, which shows a positive correlation with the rainfall intensity-ephemeris curve integral. Therefore, the runoff coefficient is a time-varying function related to the rainfall intensity and rainfall ephemeris. Based on the experimental measurement results, we fitted the binary relationship between the runoff coefficient and rainfall duration and intensity by interpolation. The final two-dimensional interpolation surface was obtained, and the time-varying runoff coefficient $\varphi_{tq}$ could be determined for a specific rainfall input condition.

#### 2.3.2. Seasonal Runoff Coefficient (SRC)

Experiments have confirmed that the process of runoff yield is influenced by multiple factors, such as latitude, climate zone, monsoon, and season. The change in air humidity, air pressure, and temperature brought on by the change of seasons will directly affect the water content in the air and soil. In the dry season, the soil water content is low, infiltration is high, and rainfall is easily absorbed by the soil. The intensity and duration of rainfall in the dry season are low, making it relatively difficult to produce waterlogging. In the rainy season, the soil water content is high, and the infiltration rate is slow, so rainfall is not easily absorbed by the soil quickly. Furthermore, the high intensity and duration of the rainfall, as well as the higher frequency of the rainfall during the rainy season, makes urban flooding relatively easy to form. Therefore, this study defines the seasonal infiltration coefficient (*SRC)* (Equation (4)) to characterize the effect of seasonal differences on surface runoff coefficient. Before calculating the *SRC*, a dispersion test needs to be performed in conjunction with the historical monthly average rainfall data. The monthly average rainfall of many years is sorted, and finally, a certain proportion of rainy season, dry season, and compromise months are divided. We choose lgX as the main function for fitting, where X ∈ (1,10) fits the seasonal variation differences of the monthly average rainfall, to describe the data characteristics and patterns. The reason is that the derivative of lgX decreases when X is close to 10, which can better describe the data characteristics of the low dispersion in the dry and rainy seasons; while the derivative increases when X is close to 1, which can reflect the high dispersion characteristics of the data in the transition season. The median month of the transition month is used as the base case. Where *M* is the corresponding value of the monthly average rainfall after ranking, *α* is the regional area compensation coefficient, which is determined according to the regional climate type and historical rainfall.

(4) SRC=1+0.1α×lgM

#### 2.3.3. Soil Erosion Coefficient (SEC)

Numerous studies have shown that soil erosion is also an important factor in flooding. Furthermore, [29] was introduced the fact that the mechanism of soil erosion by heavy rainfall is closely related to the formation of flood. The land cover type data cannot reflect the changes in water storage capacity due to the erosion in real time, thus affecting the true yield flow. Here, the regional erosion area proportion β is used to correct the yield flow, so the average weighted *SEC* of the region can be defined as Equation (5).

(5) $$SEC=\frac{1}{i}\sum_{1}^{i}\frac{1}{1-\beta_{i}}$$

#### 2.3.4. Rainfall Interval Factor ($\delta_{I}$)

Rainfall intervals lead to changes in soil aridity. According to the findings, the runoff coefficient of permeable areas is closely related to the rainfall event interval [30]. In their study, the rainfall event of 100.6 mm on 5 August 2015, lasted for 3.1 days. It continued with the next rainfall event on 9 August, and the short interval caused the water in the surface not to be drained in time, thus the initial runoff coefficient increased in vain and the surface runoff volume appearing to increase significantly. Some studies also consider the effect of circumventing short intervals of rainfall on the experimental results. Between two short intervals of rainfall, the water content in the soil or surface is at a high level because the water in the soil or surface has not infiltrated or evaporated in time [31]. When rainfall occurs at this time, a surface runoff is more likely to form and produce flooding, so the rainfall interval indicator $R_{iv}$ is defined to assess this effect. The Horton infiltration equation $f=f_{c}+\left(f_{0}-f_{c}\right)e^{-kt}$ simulates the rate change of the fluid infiltration in different media. Where f is the infiltration rate, $f_{c}$ is the steady infiltration rate, $f_{0}$ is the initial infiltration rate, t is the interval time, and k is an empirical constant related to the surface medium. Therefore, the function $e^{-x}$ is introduced as the basic function to fit the rainfall interval coefficient, C is the coefficient to be estimated from each surface infiltration experiment, and *I* is the rainfall interval (Equation (6)). The experimental results showed that the occurrence of no rainfall for more than five days could be considered a subsurface drought, and the authors finally set the surface with more than seven days of prior clear days as the base condition after the experiment [32]. When calculating the runoff coefficient of a rainfall event, first consider whether the interval exceeds seven days, if so, the initial runoff coefficient is used directly, and *I* is taken as ∞. If it does not exceed seven days, the number of interval days is introduced to make corrections to the runoff coefficient. In practice, for ease of estimation, the interval time *I* can be directly formulated to fit this degree of influence in a graded manner (Equation (7)). The non-permeable surface is not greatly affected by the previous rainfall interval, and no correction is required.

(6) $$R_{iv}=Ce^{-\frac{k}{I}}$$

(7) $$\delta_{I}=\{\begin{array}{c} \varphi_{0},\;I\geq 7\\ 1.1\varphi_{0},3\leq I\leq 7\\ 1.2\varphi_{0},I<3 \end{array}$$

In addition, the effects of slope and crown interception on the runoff coefficients were also taken into account in the final integrated runoff coefficient by experimentally fitting $\theta_{sc}$. Finally, the time-varying runoff coefficient $\varphi_{tq}$ is combined with the above SEC,SRC,$\delta_{I}$, $\theta_{sc}$ to obtain the real-time comprehensive runoff coefficient (CRC) at different landcover at any time under a real rainfall event, denoted by ω (Equation (8)).

(8) $$\omega =SEC\times SRC\times \varphi_{tq}\times \delta_{I}\times \theta_{sc}$$

#### 2.3.5. Calculation of Capacity with Coupled Overwater Capacity and Pipe Volume

Due to the complex topology of the drainage network and many calculation nodes, the drainage flow has been a difficult problem in simulation. The shape of the rainwater grate within the city is overwhelmingly a flat rectangle, and the drainage capacity per unit time can be expressed as Equation (9). The distribution of the underground drainage network affects the discharge efficiency of rainwater outfalls. Due to the characteristics of water flow fluctuation and the complexity of drainage network topology, the estimation of drainage capacity or drainage flow rate has been a difficult problem in large-scale urban flooding identification studies. In this paper, a rapid assessment method of urban large-scale drainage capacity is proposed, using the overflow rate $Q_{c}$ of above-ground rainwater grates as the base quantity. Then, the regional drainage volume density (RDVD) (Equation (10)), the ratio of the total equivalent volume of the drainage to the regional ground area) is calculated as a characteristic quantity characterizing the ability of underground pipes to quickly discharge the runoff entering the stormwater outfall. Without considering the influence of the top-support effect of the drainage network overload, the regional drainage capacity (Equation (13)) can be expressed as the product of the total water cross capacity of *m* rainwater grates (WCC) (Equation (11)) and the normalized drainage capacity assessment coefficient $\varepsilon_{nor}$ (Equation (12)), which corresponds to the total holding capacity of *n* drains in the region.

(9) $$Q_{c}=\mu z\sqrt{2gh}$$

where $Q_{c}$ is the single rainwater grate drainage flow rate (m^3^/s), *μ* is the orifice flow coefficient, *z* is the drainage orifice area (m^2^), and *h* is the wellhead water flow depth (m).

(10) $$\varepsilon_{j}=RDVD=\frac{\sum_{1}^{n}\pi r_{k}^{2}l}{A}$$

(11) $$WCC=\sum_{1}^{m}Q_{cc}$$

where r is the radius of the drain with serial number *k*, l is the length of the drain, n is the number of drains in the sub-catchment, and *j* is the catchment number.

(12) $$\varepsilon_{nor}=\frac{\varepsilon_{i}-\varepsilon_{min}}{\varepsilon_{max}-\varepsilon_{min}}$$

(13) $$Q_{d}=\varepsilon_{nor}\times WCC$$

### 2.4. Flow Concentration Simulation and Calculation

#### 2.4.1. Sub-Catchment Extraction

In this study, sub-catchments were extracted as hydrodynamic calculation units, and the results of the waterlogging extent, depth, and flow velocity were calculated by hydrodynamic equations to improve the prediction accuracy and calculation efficiency. We define the catchment area threshold that can generate surface runoff of a certain scale and form a waterlogging hazard as AC (m^2^) and perform the river classification and linkage processing in ArcGIS according to AC to derive the runoff flow concentration and transfer the relationship among sub-catchments and obtain the appropriate sub-catchment distribution.

#### 2.4.2. Analysis of Water Flow Process

The water flow process is mainly based on the single flow direction D8 algorithm. It is assumed that the water flowing in a grid can only flow into the lowest of the eight adjacent grids (if there are more than two lowest adjacent grids, then one outflow is randomly selected). The algorithm is fast and responds well to the role of the topography on the surface runoff formation. At the same time, the water flow will be blocked by buildings and change directions, which will eventually affect the spatial distribution of the ponded water. In order to realistically simulate the blocking effect of the buildings on the water flow, we superimpose the building height information with the original DEM data to obtain a new DEM_BH with the building height information. After traversing the flow direction values of all grids, we can calculate the cumulative flow values of all grids in the area, which represents the number of target grids receiving upstream convergent grids [33].

#### 2.4.3. Simulation of Evolutionary Routes Based on Urban Road Network

Although each sub-catchment is divided according to the independent catchment process, the evolutionary process of waterlogging has a holistic character, and the water flow connection between each sub-catchment needs to be considered. The terrain of urban roads is generally lower than the ordinary ground in urban areas, and rainwater grates are generally set on both sides of the roads. The strong connectivity of the road network causes waterlogging to flow and spread through the lower roads in the form of surface runoff. The roads are divided into different levels and are not included in the analysis because the highway generally has a higher roadbed or adopts an elevated form, which is not prone to waterlogging. By calculating the width of the road nearest to the pouring point in the region, the hydraulic parameters used for ponding depth calculation can be obtained. The flow of water between the upstream sub-catchment and the downstream sub-catchment is considered to be connected through the road with the lowest terrain, so the road is modeled as a U-shaped open drainage channel with width w and depth *d*.

### 2.5. Waterlogging Depth Calculation

The above parameters of the runoff yield and flow concentration stage were adjusted experimentally and by reference to the literature, and the threshold setting of the reasonable interval of the parameters was carried out for the calculation of the final water accumulation depth. Multiple parameter layers are fused within the region, with sub-catchments as boundaries. The parameter layers will include a topography layer, a subsurface drainage layer, a surface cover type layer, a surface runoff coefficient layer, etc. [34]. Hydrodynamic equations can provide accurate calculations for the waterlogging runoff. In this paper, we use our algorithm for calculating the depth of a waterlogged runoff with the nearest road as the evolution path to achieve the depth and flow rate [35].

Firstly, runoff flow $Q_{ro}$ (Equation (15)) and total flow Q (Equation (14)) can be determined by the rainfall input, drainage flow ($Q_{d}$), comprehensive runoff coefficient ω, and area A. Then the hydraulic radius R is solved jointly using the Chézy formula (Chézy coefficient) and the overwater cross-sectional flow formula (Equation (16)).

(14) $$Q=Q_{ro}-Q_{d}$$

(15) $$Q_{ro}=q\omega A$$

(16) $$Q=SC{\left(Ri\right)}^{\frac{1}{2}}$$

(17) $$R=\frac{dW}{2d+W}$$

For a U-shaped canal, the boundary of the overwater cross-section is a rectangle with two heights (*d*) and one long side (*W*). *W* is the width of the nearest road, obtained from the road data layer statistics; *d* is the waterlogging depth of (m) (Equation (17)).

## 3. Case Study

### 3.1. Study Area

**Figure 4:**
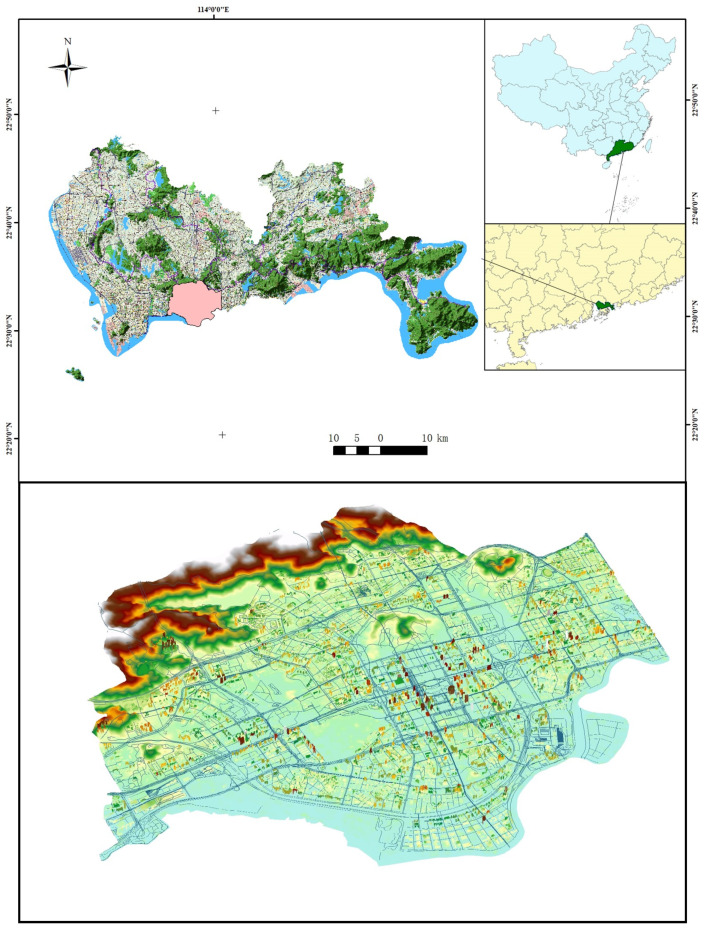
Futian District, Shenzhen, Guangdong Province.

This paper takes Futian District, Shenzhen, China, as the research object. Shenzhen, a global megacity, is one of the core cities in the Guangdong-Hong Kong-Macao Greater Bay Area (Figure 4). The population density is 8791 people per square kilometer, ranking first in China. The overall road density in the urban area is 9.50 km/km^2^, ranking first among 36 major cities in China. The higher population density and road density increases the risk of the city when facing the occurrence of waterlogging. Futian District is the central urban area of Shenzhen with a high level of development and economic density. With a resident population of over 1.66 million in 2021, its GDP accounts for 16.9% (454.6 billion yuan) of the city’s GDP (269.27 billion yuan), although its area only accounts for 4% of the city’s total area. Futian District has a total area of 78.6 square kilometers, and its topography is mainly plains, hills, mountains, and beaches, with mountains in the north and the sea in the south, and the terrain is high in the north and low in the south. Futian District is located south of the Tropic of Cancer and has a subtropical maritime climate with abundant rainfall, and an average annual precipitation of 1866 mm [36]. The rainfall is primarily concentrated from June to August, with August being typhoon season, with typhoon rainstorms and other extreme weather prone to flooding.

### 3.2. Data Processing

#### 3.2.1. Data Description

The description, sources, and accuracy of the research data used in this study are in Table 2.

#### 3.2.2. Multi-Source Data Layer Fusion Computing

The spatial extent of all data layers is bounded by the administrative division of Futian District. The rainfall input patterns are divided into the simulated rainfall and real rainfall verification. The simulated rainfall can simulate the rainfall input for different return periods, which can provide more reference for the early prevention of disasters and the identification of risk areas. Real rainfall can effectively rate the key parameters of the model and also better validate the model effect. In order to simulate the extreme rainfall event suffered by Futian district, based on the formula of the rainfall intensity (Equation (18)) published by Shenzhen Meteorological Bureau in 2015, the return periods are set as 10 years, 50 years, and 100 years, the peak rainfall is 0.4, the rainfall duration is 120 min and *τ* is set as 60 min, and the three simulated rainfall input models are obtained by using the Chicago rainfall model [37]. They correspond to the rainfall intensities of 79.434 mm/h (*a* = 10), 100.122 mm/h (*a* = 50), and 109.032 mm/h (*a* = 100).

(18) $$q=\frac{1450.239\left(1+0.594\mathrm{lg}a\right)}{{\left(t+11.13\right)}^{0.555}}$$

The real rainfall verification was selected for the rainfall event in Futian District on 11–12 April 2019. This heavy rainfall caused local waterlogging in Futian District, resulting in the drowning of three river workers due to the back-up of waterlogging. To obtain the real-time rainfall input at any given moment, we use the rainfall history data from seven weather stations located in Futian District. The rainfall trend surface was divided using Tyson polygons to obtain the rainfall input at a point.

**Figure 5:**
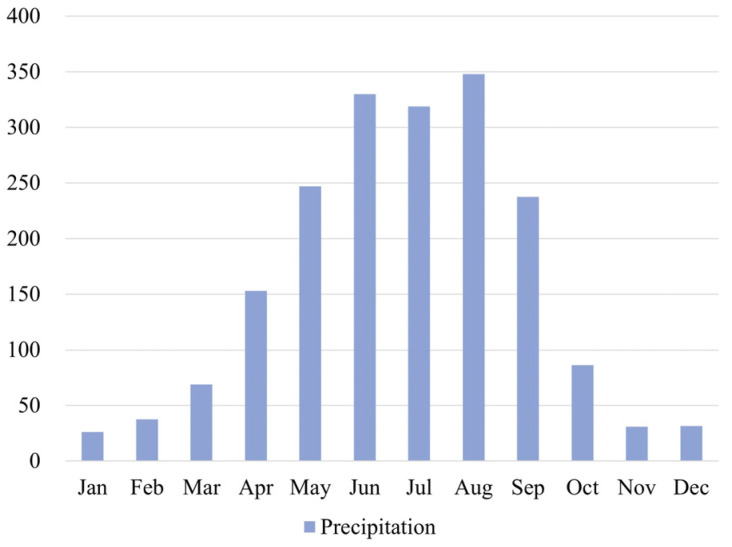
Average monthly rainfall (mm) in Shenzhen from 1961 to 2020.

By analyzing the monthly average rainfall statistics of Shenzhen for a total of 60 years from 1961 to 2020 [38] (Figure 5), the months with the most rainfall in Shenzhen are June, July, and August; the months with the least rainfall are November, December, and January, and the compromise months are February, March, April, May, September, and October. The *SRC* method was used to determine the rainy month MR (25%), dry month MD (25%), and compromise month MT (50%) for Futian District, Shenzhen.

From the statistical results (Table 3), it can be seen that the monthly average rainfall throughout the year shows a relatively obvious distribution pattern of three components. Among them, the monthly average rainfall in the rainy month MR and the dry month MD has a low degree of dispersion, with RSDs of 3.60% and 8.42%, respectively. The dispersion degree of MT in the transition month is higher, with an RSD of 58.63%. After ranking, then substitute *M* into Equation (19) to obtain *SRC* (the results are shown in Table 4). For the three storm scenarios set, a rainfall of this magnitude usually occurs in the rainy season of Shenzhen. We assume that the simulation occurs in June, July, and August, respectively, then each RC needs to be modified. The runoff coefficients of the permeable areas under the three rainfall events are assigned weighting factors 1.08, 1.09, and 1.10 [39].

(19) SRC=1+0.1α×lgM

#### 3.2.3. Runoff Yield Calculation

**Figure 6:**
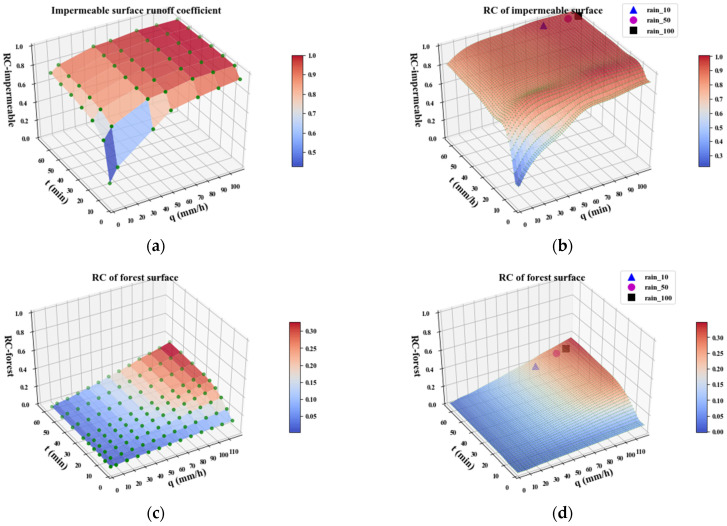

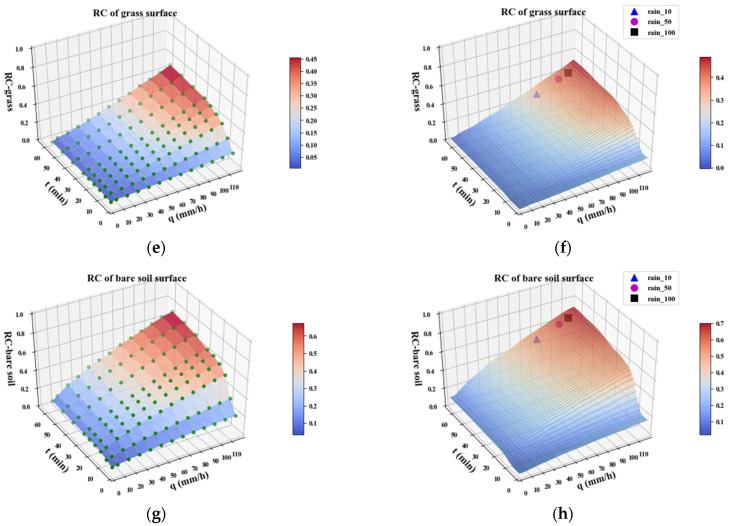
Fitted runoff coefficients of four land cover types with higher occupancy for the three rainfall input models (▲ *a* = 10, ● *a* = 50, ■ *a* = 100). (**a**,**b**): impermeable surface; (**c**,**d**): forest surface; (**e**,**f**): grass surface; (**g**,**h**): bare soil surface. Experimental values (**left**). Interpolated fitted data (**right**).

The construction land in Futian District is 5909 hectares, accounting for 74.9% of the total land area. Most of the construction land has asphalt, concrete, and cement surfaces, which have larger runoff coefficients; while woodlands, grassland, wetland, and bare soil have smaller runoff coefficients and a stronger ability to accumulate rainfall. According to the data on surface cover types, we get the range of base runoff coefficients for each material terrain [40] and use the runoff coefficients of woodland and grassland to simulate the experimental results [41,42]. As shown in (a) to (h) of Figure 6, the green dots are experimental data, varying with the rainfall intensity and rainfall ephemeris. The fitted runoff coefficients based on each surface cover type for a given rainfall intensity and ephemeral conditions were obtained by two-dimensional interpolation fitting.

**Figure 7:**
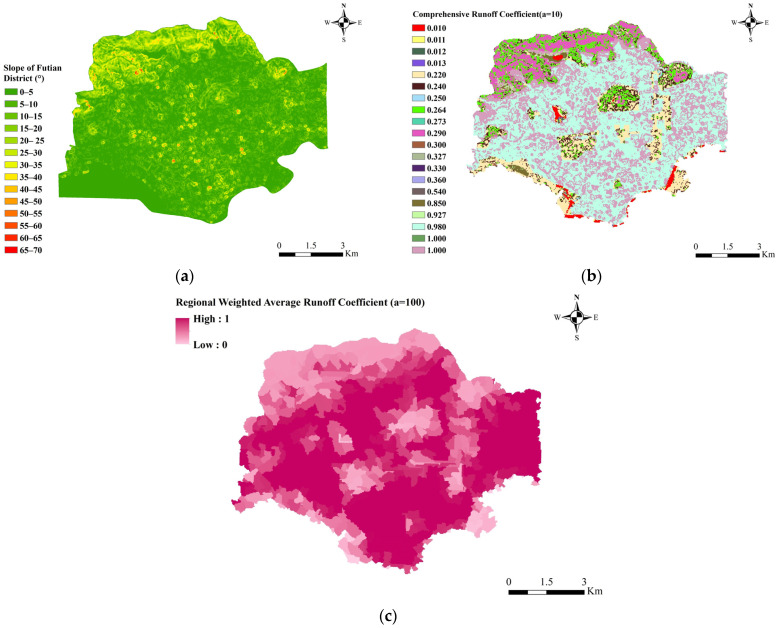
Slope distribution in Futian District (**a**), integrated runoff coefficient of each grid (**b**), weighted average runoff coefficients for each sub-catchment in Futian District (**c**).

For forested land, indices such as depression and herbaceous layer thickness were not considered here. The runoff coefficient of coniferous forests is calculated at 1.15 times that of broadleaf forests [43]. A storm scenario with *a* = 10 was used as an example to fit the real-time runoff coefficients for each grid (corresponding to different land cover types), based on the rainfall ephemeris and rainfall intensity (*t* = 60 min, *q* = 79.434 mm/h). According to the degree of quantitative influence of the slope factor on the runoff coefficient for each land cover type, the slope was classified into four classes, 0–5°, 5–10°, 10–20°, and >20°, respectively. Based on the study results [44,45], the slope-runoff coefficient multiplier relationships for permeable surfaces were fitted separately by setting the slope 0–5° as the base case, as shown in Table 5. The slope runoff coefficients were calculated by calculating the slope case (Figure 7a) for the Fountain District (Figure 7b).

According to Shenzhen 2020 soil erosion statistics data, the erosion area of Futian District is 0.75 km^2^, the total area is 78.59 km^2^, and the erosion ratio β is 0.95%, then SEC=1/ (1−β) = 1.01. The rainfall interval is set to be greater than seven days, and *I* is ∞ that is, the original runoff coefficient is taken. The *SEC* and rainfall interval factor are substituted into the runoff coefficient values of each grid in (Figure 7b), and the average runoff coefficient of a sub-catchment is calculated by zoning statistics and weighting (Figure 7c).

**Figure 8:**
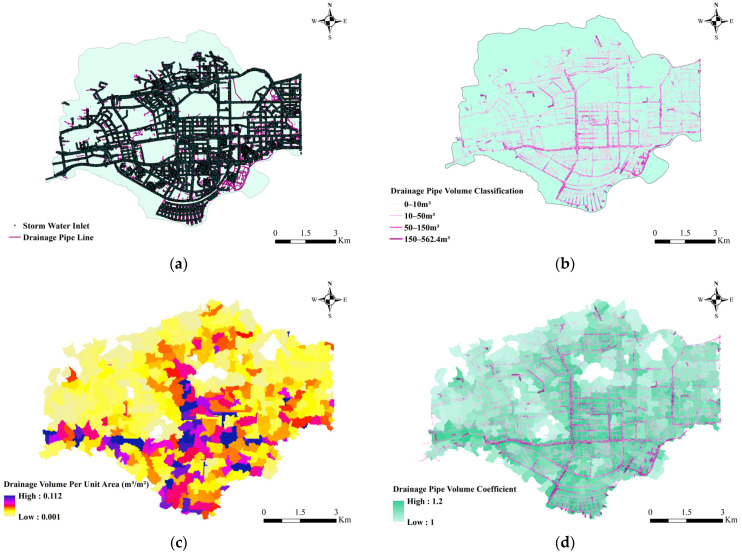
Distribution of drainage system and calculation results of drainage capacity in Futian District. (**a**) Rainwater grate and drainage pipe distribution. (**b**) Drainage pipe volume classification. (**c**) Drainage volume per unit area (m^3^/m^2^). (**d**) Drainage pipe volume coefficient.

For the discharge volume calculation, the 37,987 rainwater outfalls and 106,193 sections of drainage pipes in Futian District, Shenzhen, are mainly distributed on the surface and underground in the built-up area of the city (Figure 8a). The analysis shows that most of the rainwater outfalls are rectangular flat grates (size 75 cm × 40 cm, area 0.3 m^2^). Due to the late construction in Shenzhen, the planning and construction of pipelines are strictly based on the municipal drainage standard specifications, so it is assumed that the area with the lowest pipe density can also basically meet the daily drainage needs. The real volume of each section of drainage pipes in the catchment area was calculated based on pipe diameter and length, and the thicker line in Figure 8b represents the larger volume of that section of drainage pipes. The RDVD was calculated using the surface density of the drainage pipes (Figure 8c). The RDVD was normalized to reflect its contribution to the enhanced discharge effect at the outfall among different regions, and the amplification effect interval was set to 1.0-1.2 under the rainfall input conditions of the case (Figure 8d).

#### 3.2.4. Flow Concentration Calculation

**Figure 9:**
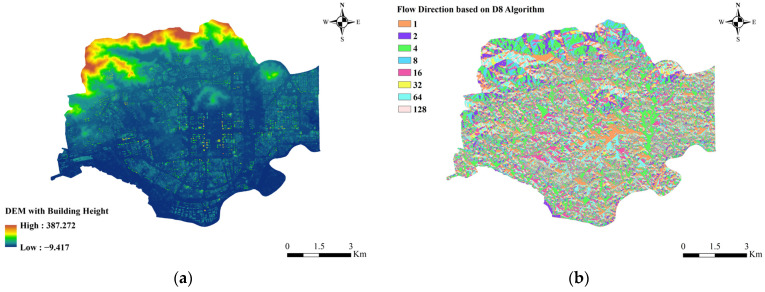
(**a**) DEM of superimposed building heights in Futian District; (**b**) Flow direction values of each grid in Futian District.

**Figure 10:**
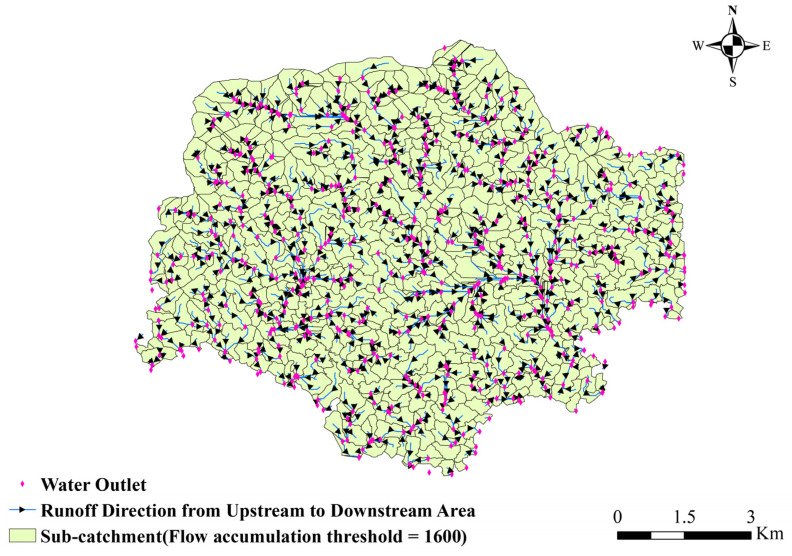
Runoff connectivity between the 596 sub-catchments.

Based on the DEM of the superimposed building height in Figure 9a, the flow direction values of each grid are calculated using the D8 single flow direction algorithm to obtain the flow direction results in Figure 9b. From the flow direction results of each grid, the cumulative value of flow is calculated. After several trials, we defined the catchment area threshold AC in Futian District that can generate the surface runoff as 40,000 m^2^. The reason is that the topography of Futian District is mostly flat, and most of the mountains are concentrated in the northern region, where the runoff flow kinetic energy is small. When it is smaller than AC, it is difficult for the area to form large-scale waterlogging in reality. A catchment area of 40,000 m^2^ corresponds to a cumulative flow value of 1600. In total, 596 sub-catchments are shown in Figure 10 after calculation and correction.

**Figure 11:**
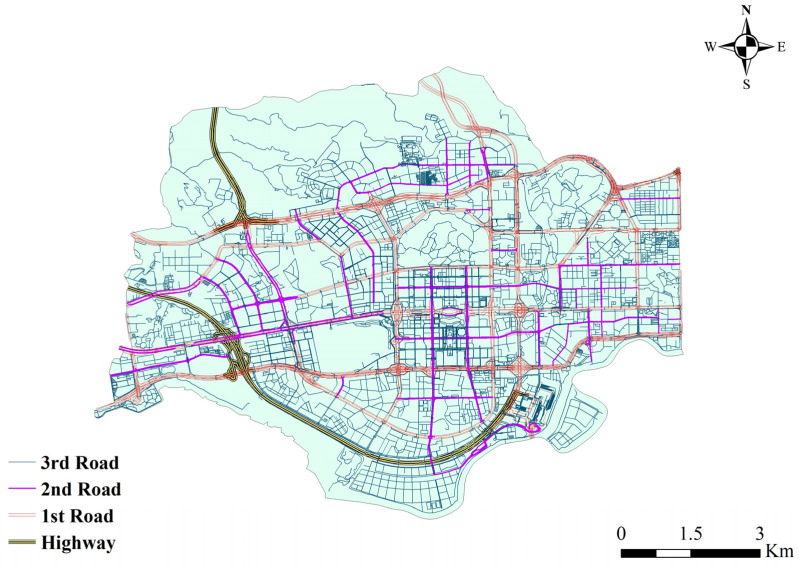
Statistics of urban road classification in Futian District.

For the surface runoff road evolution simulation, there are 166 sections of highways (2.79%), 782 sections of primary roads (Class 1 roads: national roads, provincial roads, and other main roads), 326 sections of secondary roads (Class 2 roads: ordinary main roads), and 4,675 sections of tertiary roads (Class 3 roads: town streets, rural roads, etc.) in Futian District (Figure 11), and Table 6 shows the road statistics. The regional pouring points were extracted through the connectivity relationship between each sub-catchment, and the urban roads with the closest distance to each pouring point were captured for matching. Statistically, 596 sub-catchment dumping outlets were matched to 101 (16.95%) for the Class 1 roads, 42 (7.05%) for the Class 2 roads, and 453 (76.01%) for the Class 3 roads. According to the Chinese road design specification (GB50220-95), the average widths, w, of the Class 1,2,3 roads were set at 25 m, 14 m, and 10 m, respectively.

### 3.3. Results

**Figure 12:**
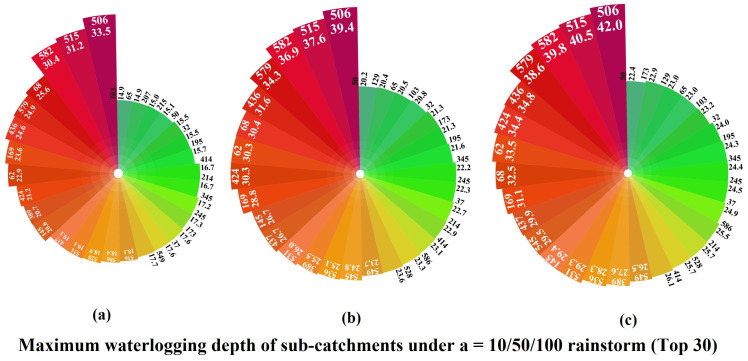
Top 30 sub-catchments with maximum ponding depth values (cm) under the three rainfall models. (**a**): *a* = 10; (**b**): *a* = 50; (**c**): *a* = 100.

**Figure 13:**
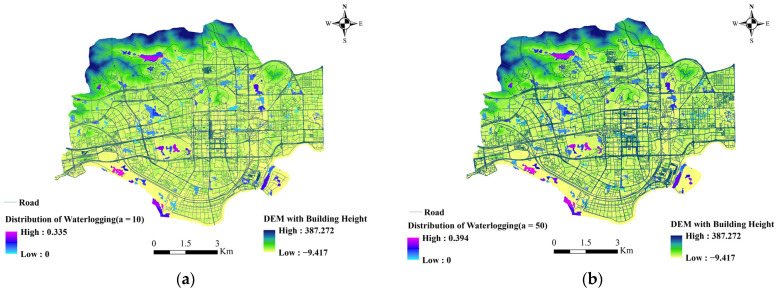

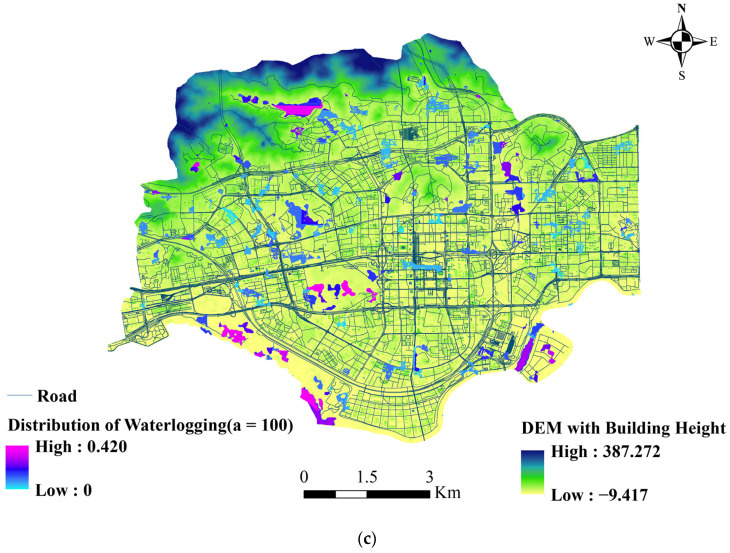
Waterlogging distribution under three rainfall input models. (**a**) *a* = 10; (**b**) *a* = 50; (**c**) *a* = 100.

The extent and depth of ponding in the 596 sub-catchments differed under the three rainfall scenarios, where the water depth values greater than 0.02 m were set as waterlogging. As shown in Table 7, in the once in 10 years rainstorm, there are 215 sub-catchments with waterlogging, the deepest depth in each area is 0.089 m, the deepest depth is 0.335 m, and the average depth of waterlogging in the whole area is 0.110 m, with a standard deviation of 0.077. In the once in 50 years rainstorms, there are 260 sub-catchments with waterlogging, the deepest depth in each area is 0.127 m, the deepest depth is 0.394 m, the average depth of waterlogging in the whole area is 0.123 m, and the standard deviation is 0.094. In the once in 100 years rainstorm, there are 277 sub-catchments with waterlogging, the average value of the deepest depth in each area is 0.146 m, the deepest is 0.42 m, the average depth of waterlogging in the whole area is 0.127 m, and the standard deviation is 0.102. As shown in Figure 12, the water depths of 582, 505, and 506 are always the highest among the top 30 sub-catchments in terms of the waterlogging depth. The other areas have smaller differences in waterlogging depths. These three areas are low-lying and adjacent to hills, which are more likely to generate flow concentration. This phenomenon is exacerbated by the lack of drainage capacity. As can be seen from the map of the waterlogging distribution in Figure 13, the average depth and extent of the waterlogging are gradually increasing as the rainfall return period increases. When the once in 100 years rainstorm occurs, a total of 5.1 km^2^ of the region will become waterlogging areas.

**Figure 14:**
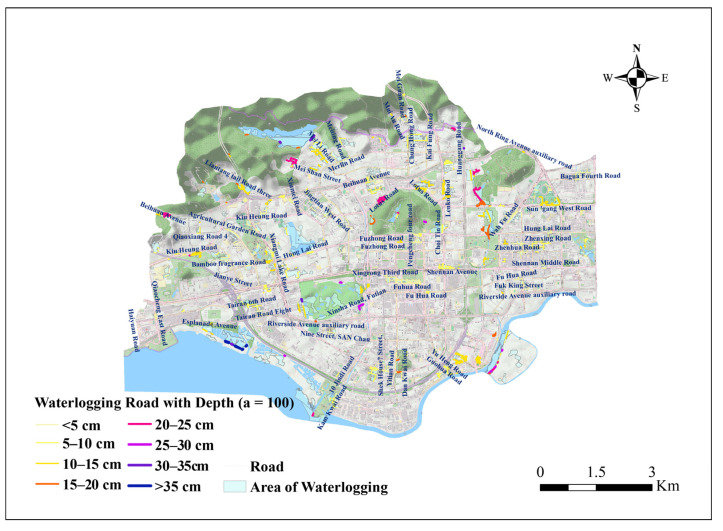
Waterlogging roads with different depth in *a* = 100 rainstorm scenario.

For road access risk, there is no unified grading standard for the road waterlogging depth. By analyzing the spatial relationship between urban roads and waterlogging areas, based on the height of the car engine intakes and the lower edge of car doors, we believe that waterlogging road sections with a grade of orange (15–20cm) or higher in the figure can have a significant impact on the traffic passage, including slowing down the vehicle passage or restricting some vehicles from passing. The road sections above 20 cm (purple and dark blue) may cause temporary or prolonged traffic disruptions by causing vehicles to stall and water to enter the vehicle. These will have an impact and change the road access options, emergency rescue dispatch, and disposal decisions (Figure 14).

In this study, the sudden rainstorm event in Futian District on the evening of 11 April 2019, was selected for truth verification. According to the data from the monitoring station 106 of the Shenzhen Water Bureau and the G3634 observation site of the Shenzhen Meteorological Bureau, the short-time intense rainfall (166 mm) brought about a sharp change in the water level of the surface ponding. From 21:00 to 21:40 (GMT+8) of the same day, the water level at this site rose from 0 to 26.0 cm within 40 min (Table 8). In addition, 106 is located in sub-catchment 479. *n* = 0.2, *J* = 5.1%, and *Q* = 1.12 m^3^/s. The depth of waterlogging at this station was obtained as 25.3 cm by hydrological calculation (*R* = 0.241, *v* = 0.437 m/s), which is closer to the sensor monitoring value (absolute percentage error: 2.7%).

## 4. Discussion

As can be seen from the waterlogging distribution maps in Figure 14, the urban inundation is usually concentrated in low-lying areas. For mountainous areas, the steep terrain makes the runoff coefficient larger than that of the flat areas. The drainage facilities in the mountainous areas are not well developed and correspond to insufficient drainage capacity. A large amount of rainfall that is not absorbed by the ground is converted into the surface runoff that moves down the slopes and collects in the low-lying areas at the foot of the mountains. In contrast, the urban built-up areas have relatively good drainage facilities, and the accumulated rainfall can usually be discharged into the underground drainage network in a timely manner, so these areas are not prone to waterlogging. However, there are some urban areas where the drainage capacity is insufficient to meet the needs of waterlogging prevention and drainage. In response to the above results, urban municipalities should strengthen the construction of drainage network facilities in the waterlogging areas, especially in the flooded areas adjacent to hills, such as by changing the drainage pipes to larger diameters and increasing the above-ground rainwater outfalls. This can enhance the ability to resist the occurrence of waterlogging when hit by typhoon and rainstorm disasters. For areas where it is difficult to change the structure of the drainage network, it is recommended to improve the waterlogging disaster plan, such as by deploying emergency drainage teams in advance to carry out emergency drainage operations on the roads, communities, and facilities with serious waterlogging, so as to reduce the impact of waterlogging disasters. Some roads with deep water may also face problems such as poor drainage (low terrain is prone to overflow), and the depth in reality may be higher than the simulation value. In extreme rainstorm scenarios, a broken vehicle trapped in standing water will be at risk of being gradually submerged over time [46]. The occupants of the vehicle need to escape in time.

Compared with other studies on waterlogging prediction and risk identification in recent years, the proposed method is more comprehensive and closer to the actual situation, and the model performance has improved (Table 9). The method has the following advantages:

(1) The runoff coefficient is an important parameter for calculating the surface runoff, which is mainly determined by the land cover type, but is also influenced by the rainfall intensity, rainfall ephemeris, season, and surface water content [48]. In practice, the runoff coefficients are supposed to be time-varying variables influenced by multiple factors. In this study, the time-varying runoff coefficients of impervious surfaces, woodlands, grasslands, and bare soils were fitted for different rainfall inputs and seasonal factor scenarios using experimental results from other studies. The surface runoff coefficients can be quickly obtained using the runoff coefficient fitting function (Figure 6).
(2) Both cities and water flows are complex systems driven by multiple factors. There is no clear demarcation between the runoff yield and flow concentration in the urban waterlogging formation mechanism [49]. The variables from multiple influencing factors need to be extracted to support the simulation process. In this study, based on a combination of references, expert experience, and historical sensor data analysis, 12 factors such as rainfall intensity, rainfall duration, rainfall interval, historical rainfall statistics, soil erosion rate, DEM, building height, surface slope, land cover type, rainwater grate, drainage pipes, urban roads, etc., are transformed into environmental variables that can be calculated. The influencing factors and variables analyzed are more comprehensive and more consistent with the real urban waterlogging formation mechanism.
(3) The quantitative estimation of large-scale drainage flow is more difficult due to the complexity of the pipe network topology and the fluctuating characteristics of the water flow. The simulation of the flow runoff yield in urban areas with a high proportion of non-permeable surfaces must consider drainage flows. The two-layer drainage capacity assessment model proposed in this paper integrates the surface layer represented by the stormwater grate with the subsurface layer represented by the drainage network. Using the overflow capacity of the surface rainwater grate as the basis for the calculation, the difference in the drainage capacity between regions is simulated by converting the volume per unit area of the subsurface drainage network (i.e., the regional drainage volume density, RDVR) into a normalized coefficient. However, because the actual flow monitoring data of the drainage pipes is missing, only a preliminary quantification of the RDVD is made here, and the parameter settings are relatively conservative. In future studies, more accurate drainage flow simulation can be achieved by extracting the characteristics of multi-region drainage networks and combining some of the actual flow measurement data.
(4) For large-scale waterlogging risk identification studies, improving the accuracy and computational speed has been the goal pursued by researchers. In this study, the parameters related to geographic factors were obtained after preprocessing the raw data using ArcGIS. The parameter layer was directly called as model input using Python to perform the hydrodynamic calculations and output the results of waterlogging depth. The accuracy of the model is verified by the true value validation in the article, and the prediction accuracy of the waterlogging depth can reach 97.3% when compared with the real rainfall event. The final absolute error is only 0.7 cm, and the relative error is 2.7%. To simulate the results for different rainfall scales in the study area, only the time-varying input variables of the parameter layer need to be adjusted, which simplifies the modeling process and improves the computational efficiency. We calculated the waterlogging depth and area of 596 sub-catchments in 2.13 s by inputting the processed parameters into the computational model under the hardware conditions of 8 core Intel (R) Xeon (R) W-2123 3.60 GHz CPU, 64.0 GB RAM and NIVDIA Quadro RTX4000 GPU.

## 5. Conclusions

The results of this study provide suggestions for the government to rapidly and effectively delineate the risk control areas, timely release the early warning information, and thus dramatically reduce or even avoid casualties and property damage due to urban waterlogging. Traffic dispatching departments can rely on this to predict the waterlogged areas in advance and release real-time information to citizens about the impact of the disasters on each road. It provides a scientific basis for navigation service providers to improve their algorithms in disaster scenarios by circumnavigating waterlogging roads in advance, based on real-time disaster information and providing more reasonable and efficient navigation path solutions. At the same time, it provides emergency dispatching departments with disaster assessment references, enhances the allocation of rescue forces in high-risk areas, conducts quantitative assessments of the accessibility of rescue services in key areas, and optimizes the layout of rescue stations and rescue force dispatching plans.

In this paper, meteorological information, geographic information, and municipal engineering information are deeply fused by multi-source fusion technology. The MDF-H large-scale waterlogging risk identification framework is proposed to realize the hydrological analysis and prediction process of horizontal evolution after the conversion of rainfall into surface runoff. Combined with the regional hydrological analysis results, 596 irregular sub-catchments are divided, and sub-catchments are set as the calculation units. The globalized unified data index and scale are adopted, while the localized data factors within the sub-catchment calculation units are fused to take into account the model’s calculation speed and accuracy. The road network at all levels in the city realizes the surface runoff linkage effect among the sub-catchments by using the hydrodynamic equation, and, finally, the information of waterlogging extent and depth in each area is output, and a real-time waterlogging distribution map is formed.

## Figures and Tables

**Table 1:** Comparison of the main methods of current waterlogging risk identification.

Indicators	Statistical Methods	Data-Driven Approach	Remote Sensing Methods	Hydrodynamic Methods	Multi-Source Data Fusion Methods
Large-scale study applicability	△△△	△△	△△△△	△	△△△△
Modeling workload	○○	○○○○	○○	○○○○○	○○
Accuracy requirements for urban subsurface data	○○○	○○	○	○○○○○	○○
Waterlogging process simulation capability	△	△	△	△△△△△	△△△
Accuracy requirements for historical flooding data	○○○	○○○○○	○○○○○	○○	○
Calculation speed	△	△△△△	△△	△	△△△△
Interpretability	△△	△	△△	△△△△△	△△△△
Prediction or identification accuracy	△△	△△△△	△	△△△△△	△△△

△: Positive indicators; ○: Negative indicators.

**Table 2:** Data sources and data resolution accuracy corresponding to each factor involved.

Item	Data Description	Data Source	Resolution
Digital Elevation Model (DEM)	Realize digital simulation of ground terrain through limited terrain elevation data (2020).	BIGEMAP	5 m × 5 m
Land cover type	Current status of all land use in the city, including construction land, broad-leaved forest land, coniferous forest land, water bodies, wetlands, etc. (2015).	2015 Global Fine Land cover product (GLC_FCS30-2015) produced by The Academy of Aerospace Information Innovation, Chinese Academy of Sciences	30 m × 30 m
Building height	Building vector files containing building location, height, number of floors, floor space, etc. (2018).	BIGEMAP	0.01 m
Drainage system	Rainwater outlet vector file, including location, orifice size, orifice shape (2015).	Water Bureau of Shenzhen Municipality (WBSM)	0.001 m
Rainfall intensity	Rainfall per unit time (2015).	Shenzhen Meteorological Bureau (SMB)	0.01 mm/min
Historical waterlogging sensor data	Waterlogging sensor monitoring data (184 stations, 2020).	Water Bureau of Shenzhen Municipality (WBSM)	0.01 m, 5 min
Historical meteorological station data	Meteorological observation data of rainfall, wind speed, visibility, temperature, and humidity at 242 stations in the city (2020).	Shenzhen Meteorological Bureau (SMB)	0.1 mm, 5 min
Urban Road network	Vector file of roads at all levels in Futian District, Shenzhen (2021).	OpenStreetMap	0.01 m

**Table 3:** Relative standard deviation (RSD).

Statistical Indicators	MR	MT	MD
MEAN	332.33	138.42	29.50
Std Dev	11.95	81.16	2.48
RSD	3.60%	58.63%	8.42%

**Table 4:** Monthly rainfall ranking and corresponding *SRC*s in Futian.

	Rank	1	2	3	4	5	6	7	8	9	10	11	12
Month													
Month		August	June	July	May	September	April	October	March	February	December	November	January
Rainfall (mm/month)		348	330	319	247	237.5	153	86.5	69	37.5	31.5	31	26
*M*		10	8	6	4	2	1	1	1/2	1/4	1/6	1/8	1/10
*SRC*		1.1	1.09	1.08	1.06	1.03	1.0	1.0	0.97	0.94	0.92	0.91	0.90

**Table 5:** Effect of slope and rainfall intensity on runoff coefficient.

Rainfall Intensity (mm/h)	0–5°	5–10°	10–20°	>20°
45~75	1.000	1.043	1.174	1.287
75~105	1.000	1.087	1.196	1.316

**Table 6:** Statistics of roads at all levels in Futian District, Shenzhen.

Indicator	Highway	Class 1 Roads	Class 2 Roads	Class 3 Roads
Number of road sections	166	782	326	4675
Total mileage	42.48 km	311.60 km	136.17 km	843.07 km
Mileage percentage	3.19%	23.37%	10.21%	63.23%

**Table 7:** Statistical results of water accumulation depth under three rainfall models.

Indicator	*a* = 10	*a* = 50	*a* = 100
Number of waterlogging areas	215	260	277
Area of waterlogging (km^2^)	3.590	4.560	5.100
Maximum depth (m)	0.335	0.394	0.420
Maximum depth mean (m)	0.089	0.127	0.146
Global average depth (m)	0.110	0.123	0.127
Standard deviation of depth	0.077	0.094	0.102
Global average flow velocity (m/s)	0.930	1.090	1.160

**Table 8:** Waterlogging data at monitoring station 106 in Futian District, 11 April 2019.

Date Time	Waterlogging Depth (m)
11 April 2019 21:00	0.000
11 April 2019 21:05	0.036
11 April 2019 21:10	0.065
11 April 2019 21:15	0.098
11 April 2019 21:20	0.130
11 April 2019 21:25	0.163
11 April 2019 21:30	0.195
11 April 2019 21:35	0.228
11 April 2019 21:40	0.260

**Table 9:** Comparison of results with other methods.

Study	Data Structure	Method	Influencing Factors
Abedin and Stephen, 2019 [34]	University of Nevada, Las Vegas main campus flooding on 11 September 2012.	GIS-framework for flood spatiotemporal variation (2019)	DEM, pour point, watershed boundary, storm drain inlet, flow travel time.
Mukherjee and Singh, 2019 [47]	Harris County, TX, USA	GIS-based weighted multi-criteria analysis to determine flood prone areas (2020)	Slope, elevation, soil type, rainfall intensity, flow accumulation, LULC, NDVI, and distance from river and distance from road.
Elkhrachy, 2015 [5]	Najran city, located in the southwestern of Saudi Arabia.	Flash flood map using satellite images SPOT and SRTM DEMs data (2015)	Land cover, drainage density, rainfall, soil influences, surface slope, surface roughness, distance to main channel.
Proposed method in paper	Futian, Shenzhen, China 2022	MDF-H waterlogging risk identification framework (2022)	Rainfall intensity, rainfall duration, rainfall interval, erosion situation, DEM, building height, slope, slope direction, surface cover type, regional drainage capacity, urban roads.

## Data Availability

All raw data can be provided by the corresponding authors upon request.
